# Dr. Barrett’s Statement

**Published:** 1892-08

**Authors:** 


### DR. BARRETT'S STATEMENT.

WE have neither the space nor desire to reply to the answer of Dr. Barrett to our editorial in the last number. The article in question was intended to be general in its application, and made no allusion by name to the University of Buffalo.

We must correct our correspondent in one particular. He states, "I made an attack upon the practice of granting the regular degree of the schools *sine curriculo*, upon a mere examination, and the outcome of the matter was the formation of the National Association of Dental Faculties."

Valuable as the influence of the *Independent Practitioner* was, the origin of the association named was based on a desire to organize the colleges for better government. Upon the call of four deans of colleges the original meeting was held.

We have permitted Dr. Barrett to be heard in defence, but it must be understood that the pages of this journal are not open to continue a local controversy.

